# Supplementary material for: Planar refraction and lensing of highly confined polaritons in anisotropic media
Source: Nat Commun. 2021 Jul 15;12:4325. doi: 10.1038/s41467-021-24599-3 (PMC8282686; doi:10.1038/s41467-021-24599-3)
Supplement: Supplementary file 1 — Supplementary Information [file 41467_2021_24599_MOESM1_ESM.pdf]

Supplementary Information

"Planar refraction and lensing of highly confined polaritons in anisotropic media"

J. Duan[1,2†], G. Álvarez-Pérez[1,2†], A. I. F. Tresguerres-Mata[1], J. Taboada-Gutiérrez[1,2], K. V. Voronin[3], A. Bylinkin[4,5], B. Chang[6], S. Xiao[7], S. Liu[8], J. H. Edgar[8], J. I. Martín[1,2], V. S. Volkov[3,9], R. Hillenbrand[4,10], J. Martín-Sánchez[1,2], A. Y. Nikitin[5,10], P. Alonso-González[1,2]*

[1]*Department of Physics, University of Oviedo, Oviedo 33006, Spain.*
[2]*Center of Research on Nanomaterials and Nanotechnology, CINN (CSIC-Universidad de Oviedo), El Entrego 33940, Spain.*
[3]*Center for Photonics and 2D Materials, Moscow Institute of Physics and Technology, Dolgoprudny 141700, Russia.*
[4]*CIC nanoGUNE BRTA, 20018 Donostia - San Sebastian, Spain.*
[5]*Donostia International Physics Center (DIPC), Donostia/San Sebastián 20018, Spain*
[6]*National Centre for Nano Fabrication and Characterization, Technical University of Denmark, DK-2800 Kgs, Lyngby, Denmark.*
[7]*DTU, Fotonik, Department of Photonics Engineering and Center for Nanostructured Graphene, Technical University of Denmark, DK-2800 Kgs. Lyngby, Denmark.*
[8]*Tim Taylor Department of Chemical Engineering, Kansas State University, Manhattan, KS 66506, USA.*
[9]*GrapheneTek, Skolkovo Innovation Center, Moscow 143026, Russia.*
[10]*IKERBASQUE, Basque Foundation for Science, Bilbao 48013, Spain.*

*Correspondence to: pabloalonso@uniovi.es

† These authors contributed equally: J. Duan, G. Álvarez-Pérez

Supplementary Note 1: Characterization of α-MoO_3 flakes transferred on air/SiO_2 structures by optical microscopy

Supplementary Figure 1a-c shows optical images of the transferred α-MoO_3 flakes on the air/SiO_2 structures with different geometries and sizes. The different thicknesses of the flakes can be identified by their different colors due to their thickness-dependent optical contrast. Remarkably, a precise positioning of the transferred flakes on top of the air structures (numbered as 1-4) acting as prisms or lenses can be done with our home-built transferring system[1].The surface roughness, edges, and thickness of the α-MoO_3 flakes were measured by atomic force microscopy (AFM) (Supplementary Figure 1d-f).

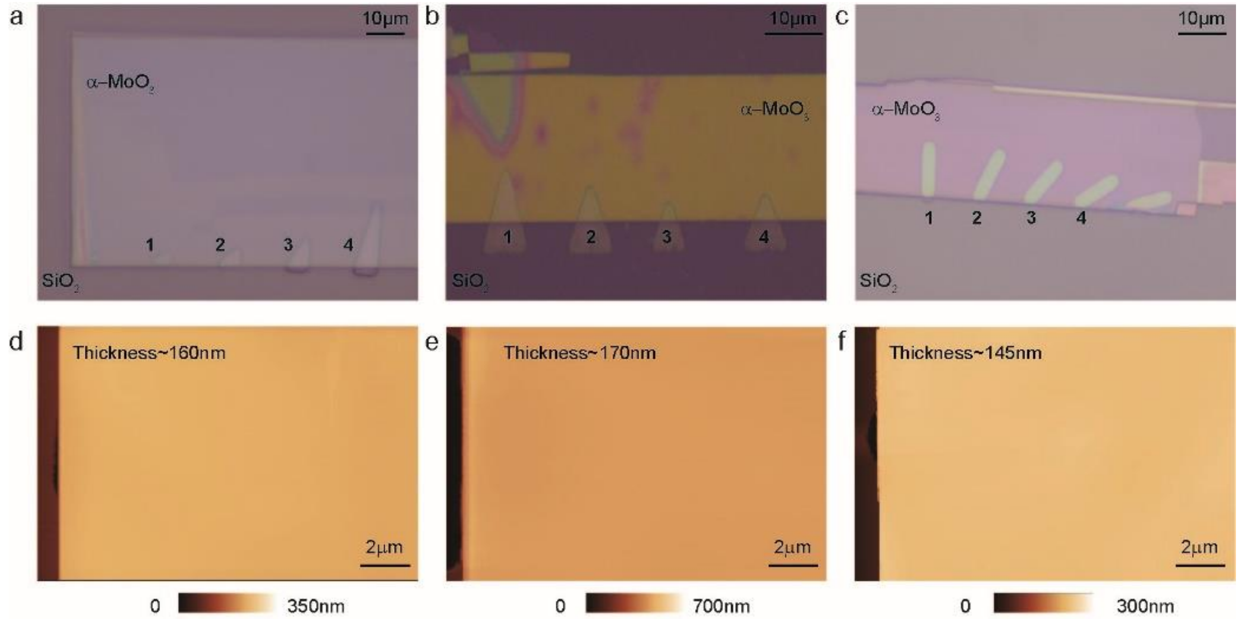

Supplementary Figure 1 Optical microscope images and AFM topography measurements of planar prisms and lenses in α-MoO_3 flakes. **a,** Optical microscopy image of a α-MoO_3 flake on top of triangular-shaped air structures acting as planar prisms. **b,** Optical microscopy image of a α-MoO_3 flake on top of triangular-shaped air structures acting as planar hyper-lenses. **c,** Optical microscopy image of a α-MoO_3 flake on top of rectangular air structures for observing double refraction of polaritons. **d-f,** AFM topography measurements of flakes in (a-c), respectively.

Supplementary Note 2: Refraction of polaritons in anisotropic media as a function of the angle of incidence and the incident wavelength

We denote the angle of refraction of the polaritonic wavevector ($\boldsymbol{k}$) and energy flux ($\boldsymbol{S}$) as $\theta_{\mathrm{out-k}}$ and $\theta_{\mathrm{out-S}}$, respectively. As discussed in Fig. 2 of the main text, the angle of refraction of polaritons propagating in anisotropic media depends on their IFC. Since the hyperbolic IFCs depend on the incident wavelength (see Fig. 2 in the main text), different angles of refraction are obtained when a structure is imaged under different incident wavelengths. This dependence is demonstrated in the columns of Supplementary Figure 2, where we vary the illuminating wavelength for different 2D prisms geometries.

Moreover, the angle of refraction is also dependent on the angle of incidence. By changing the angle at the boundary of the prisms (θ_1 / θ_2 / θ_3 in Supplementary Figure 2), the angle of incidence can be controlled, resulting in different directions of the refracted waves. The images in Supplementary Figure 2 indicate that the angle between $\mathbf{k}_{out}$ and $\mathbf{S}_{\mathrm{out}}$ becomes larger as the angle of incidence increases from ~25° to ~55°.

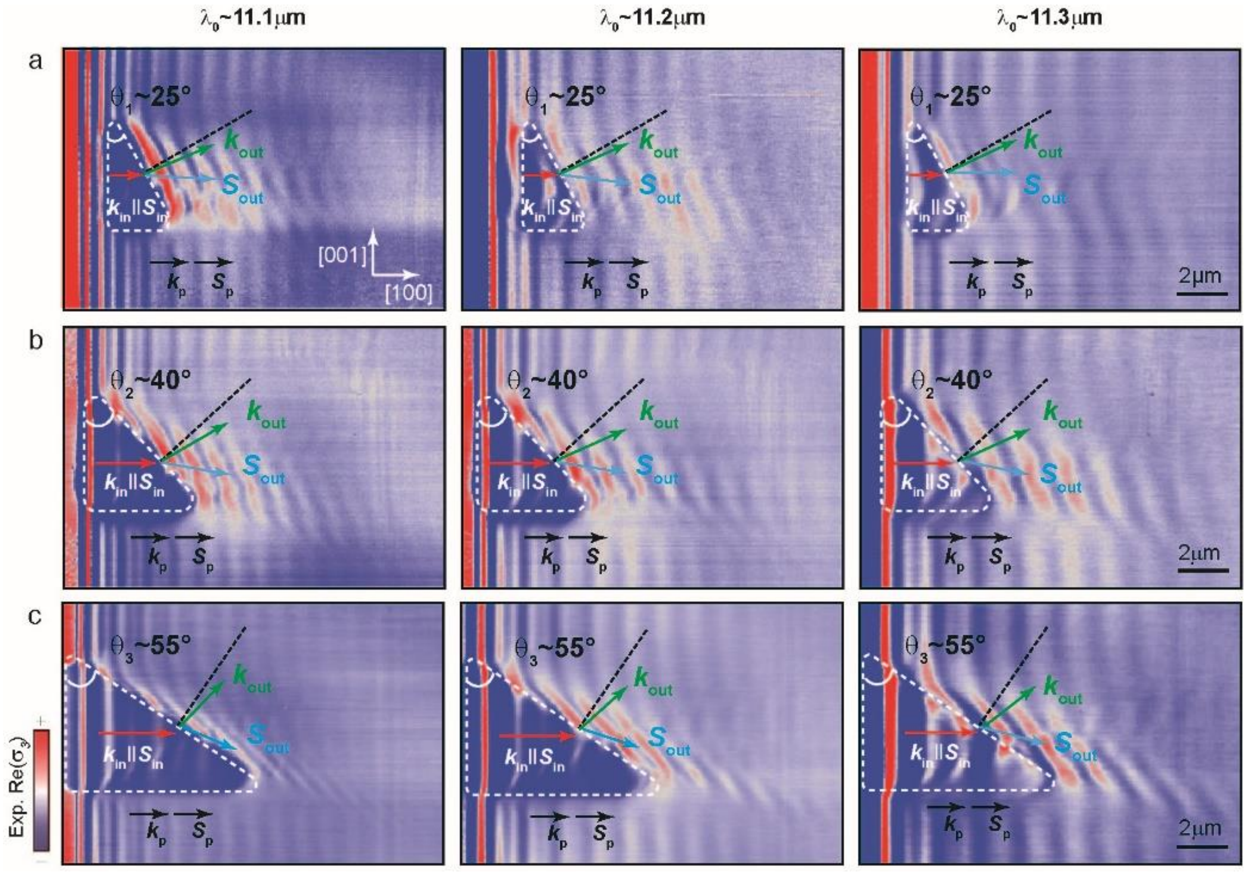

Supplementary Figure 2 Hyperbolic refraction of polaritons as a function of the angle of incidence and incident wavelength. a-c, Experimental near-field images of polaritons propagating in a 160-nm-thick α-MoO_3 flake. White dashed lines marks triangular prisms fabricated by etching an air cavity on the SiO_2 substrate below the α-MoO_3 flake. The boundary

angles are $\boldsymbol{\theta_1}$ ~ 25° (a), $\boldsymbol{\theta_2}$ ~ 40° (b), and $\boldsymbol{\theta_3}$ ~ 55° (c), respectively. The incident wavelength is 11.1 μm, 11.2 μm, and 11.3 μm in the left, middle and right panel, respectively. White arrows in the near-field image (top left) represent the crystal directions of α-MoO_3.

Supplementary Note 3: Clarification of the refractive origin of the polaritonic waves in the near-field images

Figure 2 of the main manuscript describes refraction of hyperbolic polaritons in α-MoO_3 flakes as they pass through triangular prisms, which are defined by fabricating air cavities on the SiO_2 substrates below the α-MoO_3 flakes. To rule out any possible contribution to the near-field images stemming from the excitation of polaritons by the boundaries of the air cavities, we carried out numerical simulations using large metallic prism-like structures on α-MoO_3 flakes (with the same angles as the air-cavity prisms used in Fig. 2 of the main manuscript), which act as effective metal antennas to launch polaritons in the flakes. This design resembles a potential situation where polaritons are excited by the edges of the air prisms.

However, as shown in Supplementary Figure 3, the simulated near-field images demonstrate that in the case of polaritons being launched by the metal antenna, the polaritonic waves always exhibit parallel wavefronts to the boundary of the antenna (black solid lines in Supplementary Figure 3), i.e. their wavevector (black arrows in Supplementary Figure 3) is perpendicular to the boundary. Such observation of highly confined polaritons with parallel wavefronts with respect an extended source can be explained by the Huygens' principle, which states that every point on the boundary acts as a source of wavelets that interfere, thus forming the wavefront. As the momentum of polaritons in α-MoO_3 is much larger than the size of the source, such interference gives rise to patterns that are always parallel to the contour of the source.

The behavior of polaritons launched by the edge is therefore in stark contrast to what is shown in Fig. 2, where the outcoming waves always show tilted wavefronts with respect to the boundary. As such, their wavevector not completely perpendicular to it, but tilted, which is expected by the general laws of refraction due to momentum conservation (see Fig. 1 and Fig. 2 in the main text). Hence, this difference clearly confirms refraction as the origin of the propagating polaritonic waves upon passing through the air prisms (as shown in the near-field images in Fig. 2).

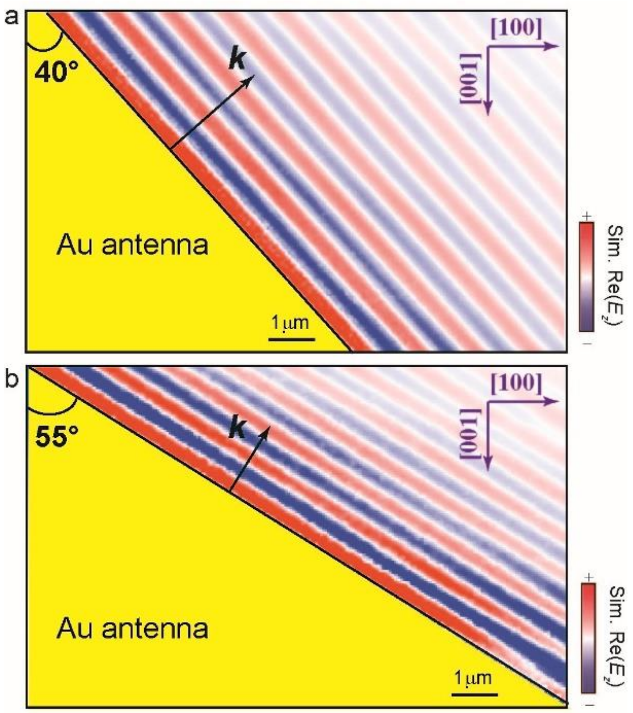

Supplementary Figure 3 Propagation of polaritons excited by gold antennas in hyperbolic media. a-b, Simulated near-field images, Re(E_z), on gold antennas placed on top of a 160-nm-thick α-MoO_3 flake whose edge is oriented at an angle of 40° (a) and 55° (b) with respect to the [001] crystalline direction in α-MoO_3. Polaritons excited by the gold antennas, with wavevector ***k*** (black arrow), exhibit wavefronts which are parallel to the boundary of the antennas in stark contrast to what is obtained in Fig. 2 of the main manuscript for refracted polaritons.

Another potential concern against the refractive origin of the polaritonic waves coming out from the prisms could be that there are hardly any visible waves inside the air-cavity prisms. Indeed, as expected in a situation in which the incident polaritons refract at each of the boundaries as they pass through the prism, the intensity of the waves inside the prisms should be much higher than that of the refracted waves (outside the prisms), due to the refraction loss at the second boundary. In this regard, we note that no clear wave oscillations are observed inside the prisms in Fig. 2 due to the selected color saturation, which was chosen to highlight and improve the visibility of the weaker refracted waves.

In Supplementary Figure 4, we show the same results as the ones depicted in Fig. 2 of the main text, but with lower saturation, where fringes ascribed to the incident waves can be clearly observed inside the prisms. Also, we would like to note that, regarding contrast in the experimental s-SNOM images, it is necessary to take into account the non-negligible near-field contribution of the tip-sample interaction (especially for metal tips, which are generally employed due to their large scattering cross-section). This contribution typically consists of a constant complex value added to the complex polaritonic signal. As in our measurements we have two effective media, α-MoO_3 /air and α-MoO_3 /SiO_2, it is expected that such contribution gives rise to small offset differences in the real part of the near-field plots in one region with respect to the other. In turn,

these differences are not present in the numerical simulations. Nevertheless, this contribution is not expected to have any influence on the direction of the polaritonic wavevectors and Poynting vectors, neither on their relative contrast, allowing us to unambiguously visualize refraction phenomena.

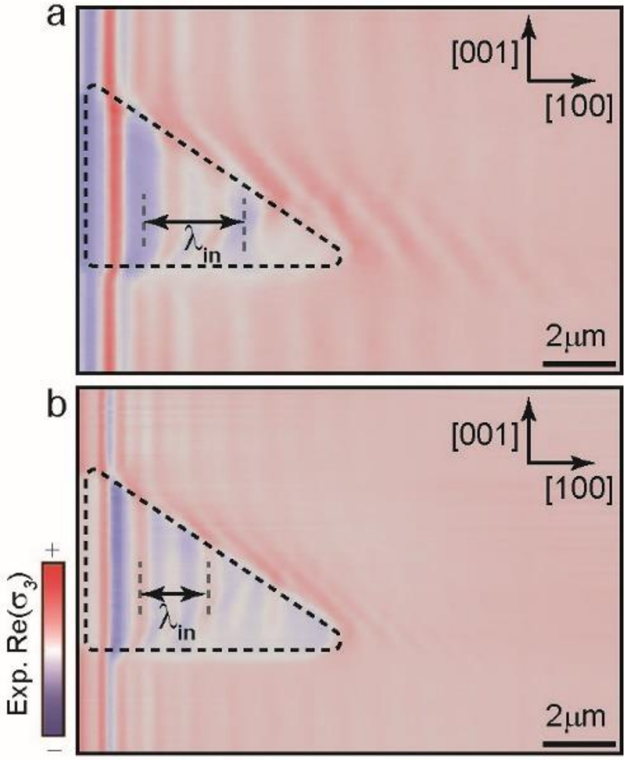

Supplementary Figure 4 Visualization of incident waves inside the prisms for refraction of polaritons in anisotropic media. a-b, The same experimental near-field images as in Fig. 2a-b of the main text, but with different color saturation. With less saturation, fringes ascribed to incident waves are clearly observed inside the prisms. The wavelength of incident waves are marked by black arrows.

Supplementary Note 4: Lensing of polaritons in anisotropic media as a function of the angle of incidence and the incident wavelength

The full-width at half maximum (FWHM) of the focus spot and the focal distance of the refractive hyperbolic lens (triangular shape), shown in Fig. 4 of the main manuscript, are determined by $\mathbf{k}_{out}$ and $\boldsymbol{S}_{out}$, respectively, which eventually depend on the incident free-space photon wavelength (λ_0) and the angle of incidence of the incoming polaritons. In Supplementary Figure 5, we show near-field images of the same lens used for Fig. 4 in the main text, but taken at different incident wavelengths. When we increase the incident wavelength from $\lambda_0 \sim 11.04\,\mu m$ (Supplementary Figure 5a) to $\lambda_0 \sim 11.08\,\mu m$ (Supplementary Figure 5b) and $\lambda_0 \sim 11.16\,\mu m$ (Supplementary Figure 5c), the hyperbolic IFCs become more open, yielding refracted polaritons with different

$\mathbf{k}_{out}$ and $\boldsymbol{S}_{out}$. Given that the focusing arises from the interference of refracted polaritons, the FWHM of the focus spot should be of the same order as the half the wavelength of the refraction polaritons ($\lambda_{out}/2$, with $\lambda_{out} = 2\pi/|\mathbf{k}_{out}|$). Meanwhile, the focal distance changes from a value of ~0.7 μm (Supplementary Figure 5a) to ~1.2 μm (Supplementary Figure 5b), and ~1.8 μm (Supplementary Figure 5c) due to the different directions of $\boldsymbol{S}_{out}$ for different incident wavelengths.

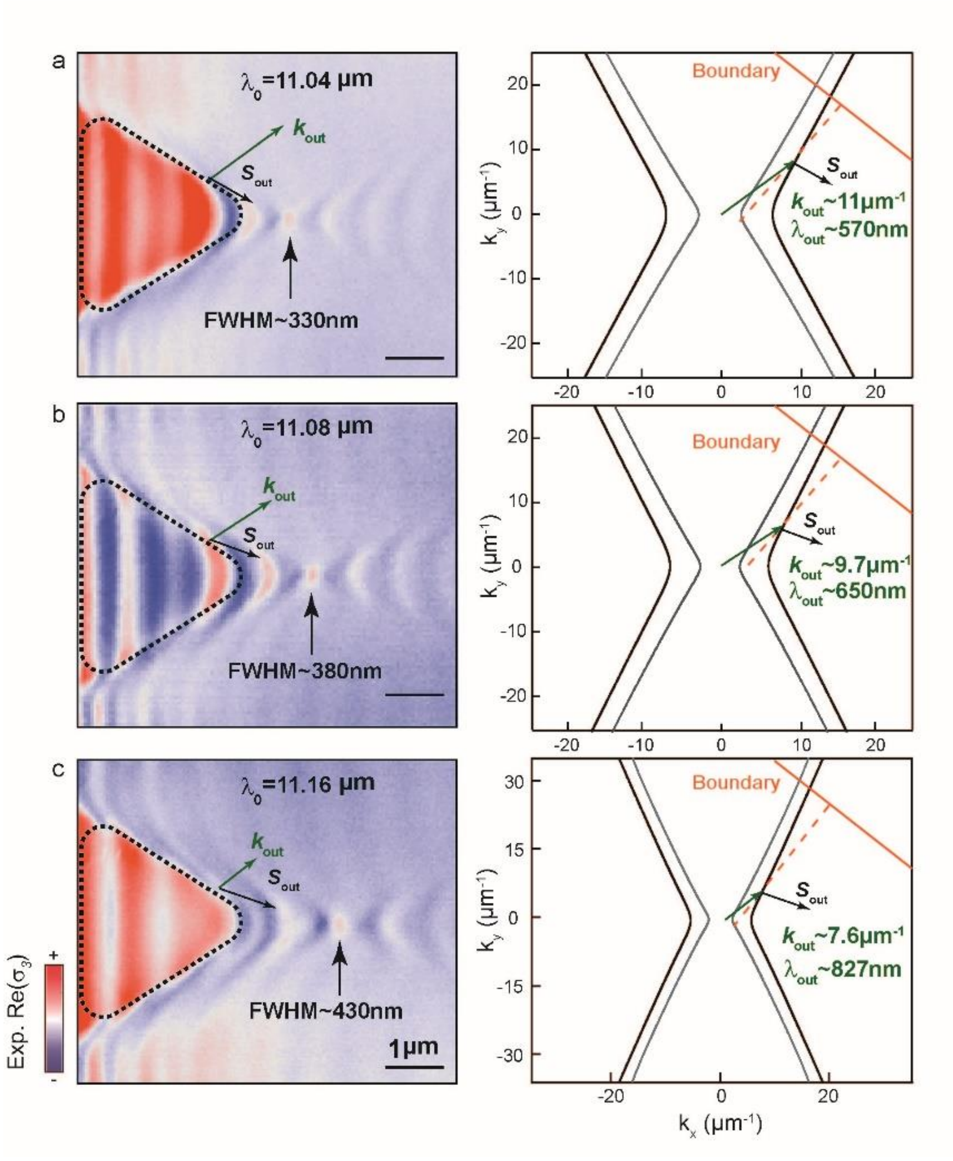

Supplementary Figure 5 HPhPs focusing in hyperbolic lenses with varying incident wavelength. a-c, (Left panel) Experimental near-field images of lensing of polaritons at excitation wavelengths λ_0 = 11.04 μm (a), 11.08 μm (b), 11.16 μm (c), respectively. By varying the incident wavelength, both the FWHM of the focus spot and the focal distance of hyperlens are controlled. **(Right panel)** The corresponding analytic IFCs and the wavelength obtained for the refracted

polaritons (λ_{out}), being approximately the double of the FWHM value for the focus spot. Grey and black curves represent IFCs of polaritons in the α-MoO_3/air and α-MoO_3/SiO_2 media, respectively.

Besides on the incident wavelength, refraction of polaritons in anisotropic media also depends on the angle of incidence of the incoming polaritons, as shown in Supplementary Figure 6. When the angle of incidence varies from θ_1~57° (Supplementary Figure 6a) to θ_2~62° (Supplementary Figure 6b), the modulus of refracted wavevector also increases (Supplementary Figure 6c), indicating that the wavelength of the refracted polaritons (λ_{out}) and the corresponding FWHM of the focus spot become smaller. In addition, the direction of $\boldsymbol{S}_{out}$ varies (Supplementary Figure 6c), which makes the focal distance to change from a value of ~2.5 μm (Supplementary Figure 6a) to ~1.3 μm (Supplementary Figure 6b).

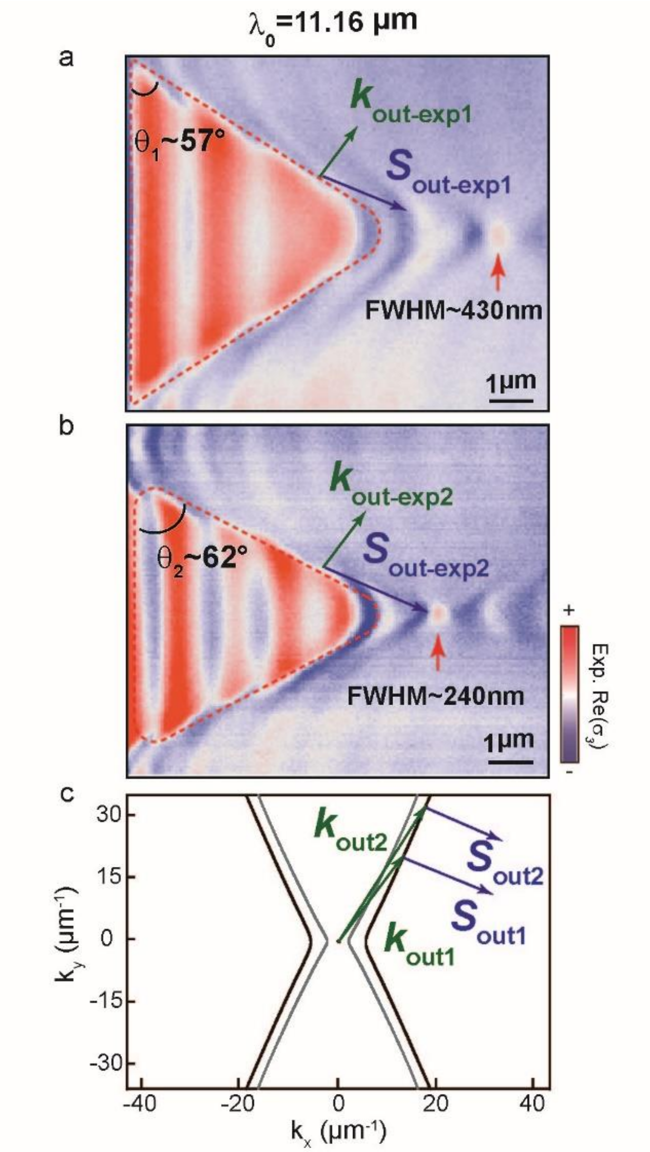

Supplementary Figure 6 HPhPs focusing in hyperbolic lenses with varying geometries. a-b, Experimental near-field images of a polaritonic hyperlens with the boundaries forming an angle

of $\theta_1 = 57°$ (a) and $\theta_2 = 62°$ (b), respectively. **c**, The corresponding analytic IFCs (grey for α-MoO_3/air and black for α-MoO_3/SiO_2) produce different wavevector and energy flux directions of the refracted polaritons for the two geometries of the lenses. These values are in good agreement with the experiments shown in (a-b).

Supplementary Note 5: Optimization of a lens for polaritons in hyperbolic media

Focusing of polaritons is obtained upon the interference of the outgoing refracted polaritons as they pass through the refractive lens (prism). The wavevectors ($\mathbf{k}_{out}$) and direction ($\mathbf{S}_{out}$) of the refracted polaritons depend on the angle of the boundaries defining the lens (θ), or equivalently, on the angle of incidence, as shown in Supplementary Figure 6. The size of the focus spot is intimately related to the wavelength of the refracted polaritons (λ_{out}, being $2\pi/|\mathbf{k}_{out}|$): smaller sizes are obtained for smaller λ_{out}. Moreover, $|\mathbf{k}_{out}|$ increases as the angle that the wavevector forms with the x axis, α, increases up to the asymptote of the hyperbolic IFC. Therefore, the polaritonic wavelength (and, therefore, the size of the focus) is smaller as we approach the angle of the asymptote of the hyperbolic IFC.

On the other hand, since polaritons decay exponentially as they propagate, the intensity at a distant focus can be rather weak. Propagation losses are more remarkable for polaritons with larger wavevectors (smaller wavelengths).

There is thus a compromise between momentum (directly related to the focus size) and propagation loss (directly related to the intensity at the focus spot). The propagation loss of polaritons can be evaluated by the value of their figure of merit (FOM), which is defined by L_p/λ_p, L_p and λ_p being the polaritonic propagation length and wavelength, respectively. It should be noted that for a given design of the lens where its boundaries are perpendicular to the asymptote of the hyperbolic IFC, the refracted polaritons possess infinitely large momentum, thus setting a lower limit to the size of the focus spot, however, with relatively high losses as mentioned above.

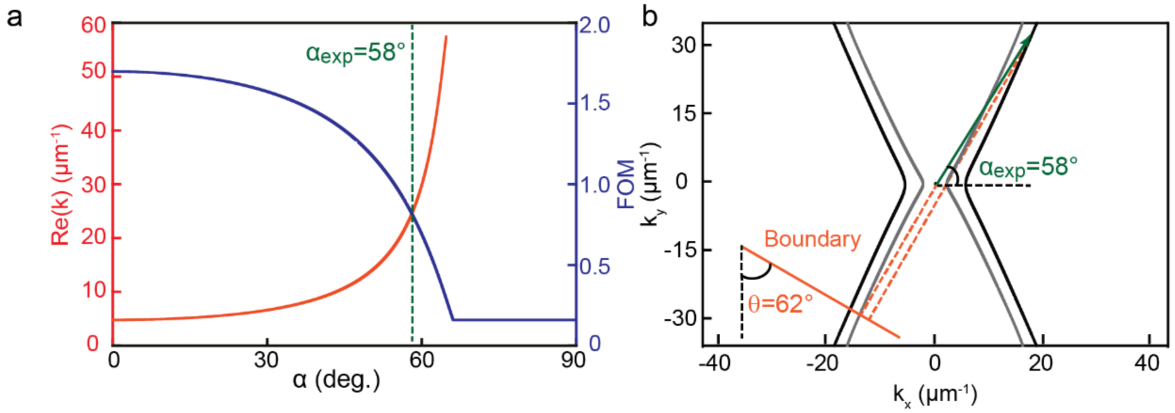

Supplementary Figure 7 Optimization of a lens for polaritons in hyperbolic media. a, Calculated modulus of the wavevector ($\boldsymbol{k}$) and the figure of merit (FOM) of polaritons in a 170-nm-thick α-MoO_3 flake as a function of the angle (α) between $\boldsymbol{k}$ and the direction of $k_y = 0$ in the IFCs. **b**, Corresponding analytic IFC of polaritons in the same α-MoO_3 flake as in (a) on top of SiO_2 (black curve) and air (grey curve).When the angle of the boundary (orange line) is chosen $\theta = 62^\circ$, we obtain an angle $\alpha_{exp} = 58^\circ$ based on momentum conservation at the boundary.

In the following, we optimize the design of the lens (angle of their boundaries, θ) for enhanced focusing of polaritons. To do that, we analytically calculate the modulus of the polaritonic wavevector and FOM as a function of the angle (α) between the wavevector (**k**) of the refracted polaritons and the direction of the x axis, i.e. the axis k_y = 0 (see Methods), as shown in Supplementary Figure 7a. The crossing point at $\alpha = 58°$ gives us an optimal value for the design of the lens, which minimizes both the focus size and the optical losses, as it is close to the asymptote of the hyperbolic IFC. The experimental results presented in Fig. 4 of the main manuscript correspond to a lens with a boundary tilted an angle $\theta = 62°$, nearly perpendicular to the asymptote of the hyperbolic IFC, which yields an angle $\alpha_{exp} = 58°$ based on momentum conservation at the boundary (Supplementary Figure 7b). Remarkably, by using the optimized design, a focus with a FWHM of ~λ_p/6, $\lambda_p$being the polaritonic wavelength along [100] crystal direction of α-MoO_3, is obtained.

Supplementary Note 6: Comparison between refraction of polaritons in isotropic and anisotropic media.

In order to compare the refractive characteristics of polaritons in anisotropic and isotropic media, we place α-MoO_3 (in-plane anisotropic) and h-BN (in-plane isotropic) flakes on top of similar air-cavity prisms built on SiO_2 substrates.

On the one hand, polaritons refract at the boundary of the α-MoO_3/air prism into the α-MoO_3/SiO_2 region along a given direction, which is given by the Poynting vector ($\boldsymbol{S}_{\text{out-exp}}$, blue arrows in Supplementary Figure 8a), with respect to which their wavefronts are tilted ($\mathbf{k}_{\text{out-exp}}$, green arrows). Thus, the Poynting vector ($\boldsymbol{S}_{\text{out-exp}}$) and the wavevector ($\mathbf{k}_{\text{out-exp}}$) of the refracted polaritons are not collinear, as already shown in Fig. 2 of the main text. The numerical simulation (middle row in Supplementary Figure 8a) perfectly reproduces this feature, mimicking the phenomenon of refraction in the experiment. Moreover, it agrees well with the theoretical prediction (bottom row in Supplementary Figure 8a), which is calculated based on both momentum conservation at the boundary and the analytical IFC (see Methods). On the other hand, the refractive characteristics are very different in isotropic media, where **k** and **S** are always collinear due to the circular IFC (Supplementary Figure 8b).

Interestingly, these different properties of polaritons refracting in isotropic and anisotropic media have profound implications. For instance, a wave passing from a low refractive index isotropic medium to a high refractive index isotropic medium bends toward the normal (Supplementary Figure 8b), in accordance to our daily experience. In stark contrast, a wave passing from a low

refractive index hyperbolic medium to a high refractive index hyperbolic medium counterintuitively bends away from the normal (Supplementary Figure 8a).

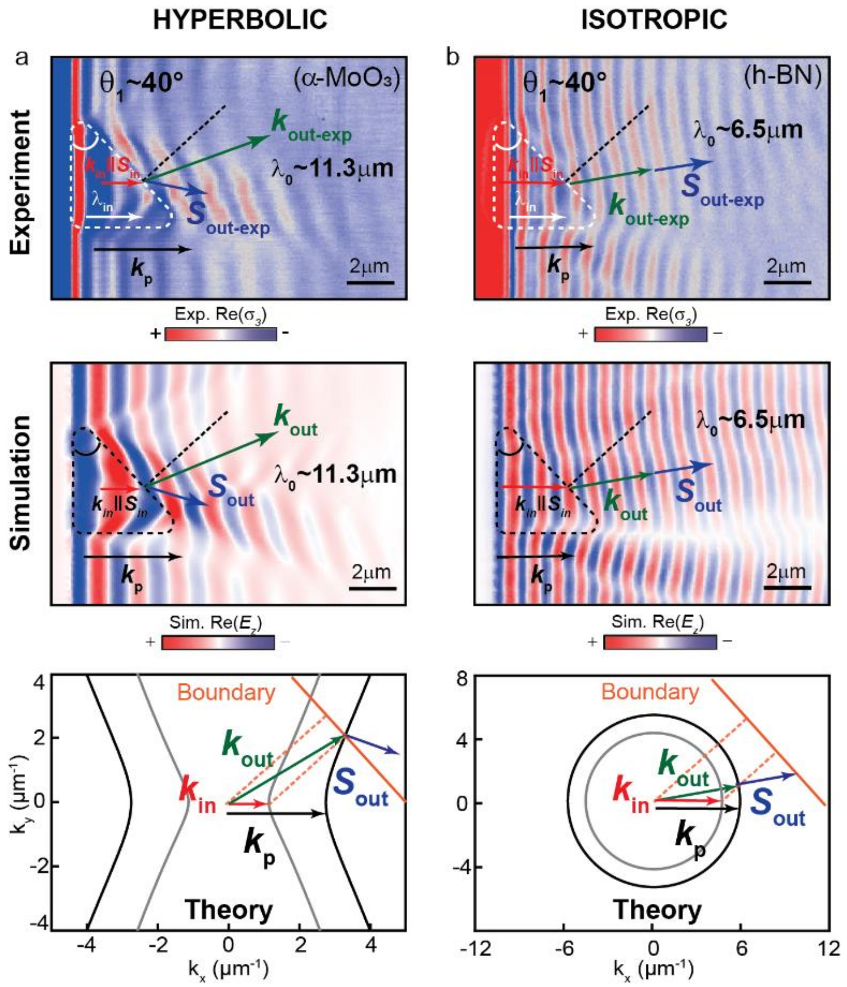

Supplementary Figure 8 Refraction of polaritons in hyperbolic and isotropic media. a, Experimental (top row) and simulated (center row) near-field images of HPhPs propagating in a 160-nm-thick α-MoO_3 flake at λ_0 = 11.3 μm. Upon refraction at the right boundary of a triangular prism (dashed triangle) with an angle θ_1~40°, HPhPs bend away from the normal, $\mathbf{S}_{\mathrm{out-exp}}$ (blue arrow), with a tilted wavevector $\mathbf{k}_{\mathrm{out-exp}}$ (green arrow). **b,** Experimental (top row) and simulated (center row) near-field image of in-plane isotropic polaritons in a 93-nm-thick h-BN flake at λ_0 = 6.5 μm. Upon refraction at the right boundary of a triangular prism (dashed triangle) tilted an angle θ_1 ~ 40°, in-plane isotropic polaritons in h-BN bend towards the normal, with collinear $\mathbf{k}_{\mathrm{out-exp}}$ (green arrow) and $\mathbf{S}_{\mathrm{out-exp}}$ (blue arrow). From the corresponding analytic IFCs (grey for the substrate as air while black for the substrate as SiO_2) and considering momentum conservation at the boundary (orange lines), the extracted $\mathbf{k}_{\mathrm{out}}$ and $\mathbf{S}_{\mathrm{out}}$ are in good agreement with experiment and simulation for both α-MoO_3 and h-BN cases.

Supplementary Note 7: Study of the focal line of planar lenses in hyperbolic media.

In isotropic media, a focal line for a 2D lens is defined as a line drawn perpendicular to the lens axis at the focal point. However, in hyperbolic media, the definition of the focal line for a lens is complex due to the non-collinearity in $\mathbf{k}$ and $\mathbf{S}$ for the incident polaritons. Here, we extract the focal line of our optimized lens based on a semi-analytical approach, in which we extract the direction of refracted polaritons ($\mathbf{S}_{\mathrm{out}}$) considering momentum conservation at the boundary of the lens, together with the analytic IFC. The position of the focus spot is obtained graphically by drawing the direction of the refracted polaritons with $\mathbf{S}_{\mathrm{out}}$ after passing through the lens.

Let us consider the two possible cases for incident polaritons in α-MoO_3 at the boundary of the lens in terms of collinearity of $\mathbf{k}_{\mathrm{in}}$ and $\mathbf{S}_{\mathrm{in}}$. These two cases are: $\mathbf{k}_{\mathrm{in}}$ and $\mathbf{S}_{\mathrm{in}}$ collinear (shown in Fig. 2 of the main manuscript) and $\mathbf{k}_{\mathrm{in}}$ and $\mathbf{S}_{\mathrm{in}}$ non-collinear (shown in Fig. 3 of the main manuscript).

For the case of incident polaritons with collinear $\mathbf{k}_{\mathrm{in}}$ and $\mathbf{S}_{\mathrm{in}}$ at the boundary of the lens (only possible along the x axis, i.e. along the direction $k_y = 0$), we study the influence of the tilting angles of $\mathbf{k}_{\mathrm{in}}$ at the boundary on the formation of a focus spot (Supplementary Figure 9) by rotating the α-MoO_3 crystal axes with respect to the boundary of the lens. In this case, the IFCs are also rotated. In such case, we find that even under a small tilting angle of $\mathbf{k}_{\mathrm{in}}$ with respect to the boundary (e.g. 5 º), refraction of polaritons is not allowed for one of the boundaries of lens (Supplementary Figure 9), and therefore no focusing is obtained. This can be better understood considering that the hyperbolic IFCs in both α-MoO_3/SiO_2 and α-MoO_3/air media are very similar, and that the angle of the boundary for our optimized lens (Supplementary Figure 7) is quite close to the asymptote of IFCs. Therefore, the existence of a $\mathbf{k}_{\mathrm{out}}$ that crosses the second hyperbolic IFC in the α-MoO3/SiO_2 medium while fulfilling momentum conservation is not possible.

For the general case of non-collinear $\mathbf{k}_{\mathrm{in}}$ and $\mathbf{S}_{\mathrm{in}}$, two scenarios are possible depending on the direction of $\mathbf{k}_{\mathrm{in}}$ with respect to the axis $k_y = 0$:

i) The tilt angle of $\mathbf{k}_{\mathrm{in}}$ takes values between ~ -35º and +35º (Supplementary Figure 10). In this case, we obtain a very interesting result: all refracted polaritons focus at the same focal point (defined by the crossing point of the $\mathbf{S}_{\mathrm{out}}$ where the refracted polaritons interfere), regardless of the tilt angle of $\mathbf{k}_{\mathrm{in}}$ (Supplementary Figure 10b-d). This effect can be understood considering that momentum conservation at the boundary yields very large refracted wavevectors $\mathbf{k}_{\mathrm{out}}$ (ending within the grey shaded area in Supplementary Figure 9a), while exhibiting the same direction as $\mathbf{S}_{\mathrm{out}}$. This is because the Poynting vector is perpendicular to the IFC, and the hyperbolic IFC for such wavevectors is almost a straight line (Supplementary Figure 9a).

ii) The tilt angle of $\mathbf{k}_{\mathrm{in}}$ is larger than +-35º, but not too large so that there is a focusing effect. In this case, momentum conservation at the boundary yields small refracted wavevectors $\mathbf{k}_{\mathrm{out}}$ that lay within the white areas in Supplementary Figure 9a, where the curvature of hyperbolic IFCs shows a non-monotonical variation. Consequently, a large variation of the angles for $\mathbf{S}_{\mathrm{out}}$ is obtained, resulting in a large variation of the spatial localization of the focus. This effect can be better observed by choosing a more open angle

for the boundary of the lens (Supplementary Figure 11), which yields, upon momentum conservation, a broader range of refracted wavevectors $\mathbf{k}_{\mathrm{out}}$ laying within the white area in Supplementary Figure 11, that is, in the region where the hyperbolic IFCs exhibit a non-monotonical curvature, resulting in a complex trajectory of the focus.

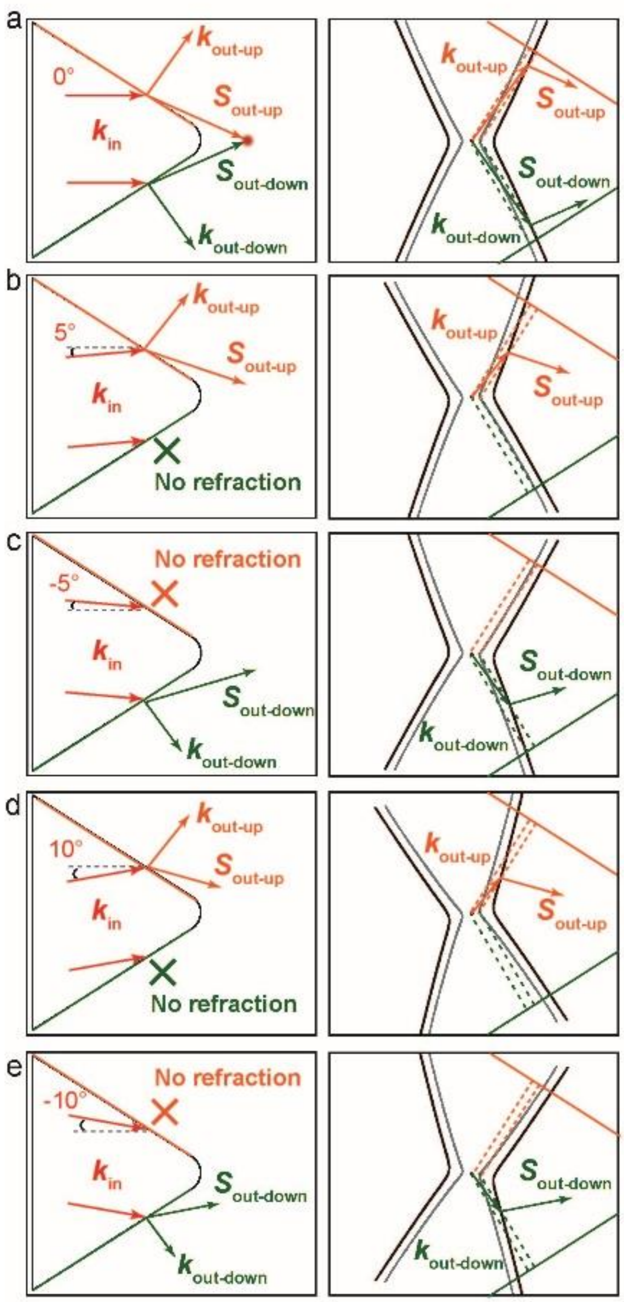

Supplementary Figure 9 Hyperlens with tilted incident waves of collinear *k* and *S*. a-e, Focusing of HPhPs with collinear incident $\mathbf{k}_{\mathrm{in}}$ and $\mathbf{S}_{\mathrm{in}}$ for a representative tilt angle at 0º (a), 5º (b), -5º (c), -10º (d), and -10º (e) based on a semi-analytical approach. The grey and black curves

in the right column represent the IFCs of polaritons in the α-MoO_3/air and α-MoO_3/SiO_2 media, respectively. Note that to obtain polaritons in α-MoO_3 with collinear $\mathbf{k}_{\mathrm{in}}$ and $\mathbf{S}_{\mathrm{in}}$ at the boundary of the lens in each case, it is necessary to rotate the α-MoO_3 in-plane crystal axes with respect to the boundary of the lens. Rotated IFCs are shown in the right column.

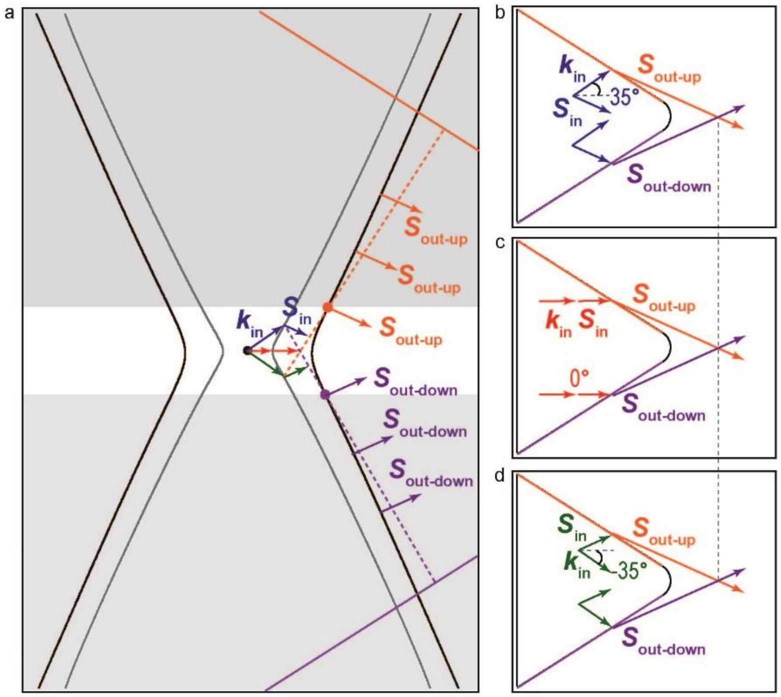

Supplementary Figure 10 In-plane hyperlens performance upon tilted incident polaritons with non-collinear k and S. a, Analytic IFCs of polaritons propagating in a 170-nm-thick α-MoO_3 on top of air (grey curve) and SiO_2 (black curve), respectively. When the tilting angle of $\mathbf{k}_{\mathrm{in}}$ takes values between ~ -35° and 35° (within the section between the violet and green arrows as $\mathbf{k}_{\mathrm{in}}$), the refracted wavevector enters the grey shaded region, in which the hyperbolic IFCs are almost straight lines yielding the same direction of $\mathbf{S}_{\mathrm{out}}$. When the tilting angle of $\mathbf{k}_{\mathrm{in}}$ is larger than +35° or smaller than -35°, the refracted wavevector enters the white region, in which the hyperbolic IFCs show a non-monotonically variation, thus yielding a large variation of angles for $\mathbf{S}_{\mathrm{out}}$ (see also Supplementary Figure 11). The orange and violet solid lines represent the upper and lower boundary of the lens, respectively. **b-d**, Focusing of HPhPs with non-collinear incident $\mathbf{k}_{\mathrm{in}}$ and $\mathbf{S}_{\mathrm{in}}$ for a representative tilting angle of 35° (b), 0° (c), and -35° (d) based on a semi-analytical approach. We extract the direction of the refracted polaritons ($\mathbf{S}_{\mathrm{out}}$) based on both momentum conservation on analytical IFCs, and we obtain the focus spot by drawing the geometries of the lenses and the crossing point of $\mathbf{S}_{\mathrm{out-up}}$ (orange arrows) and $\mathbf{S}_{\mathrm{out-down}}$ (violet arrows) graphically. All the refracted polaritons focus at the same focal point (defined by the crossing point of the different vectors $\mathbf{S}_{\mathrm{out}}$), regardless of the tilting angle of $\mathbf{k}_{\mathrm{in}}$.

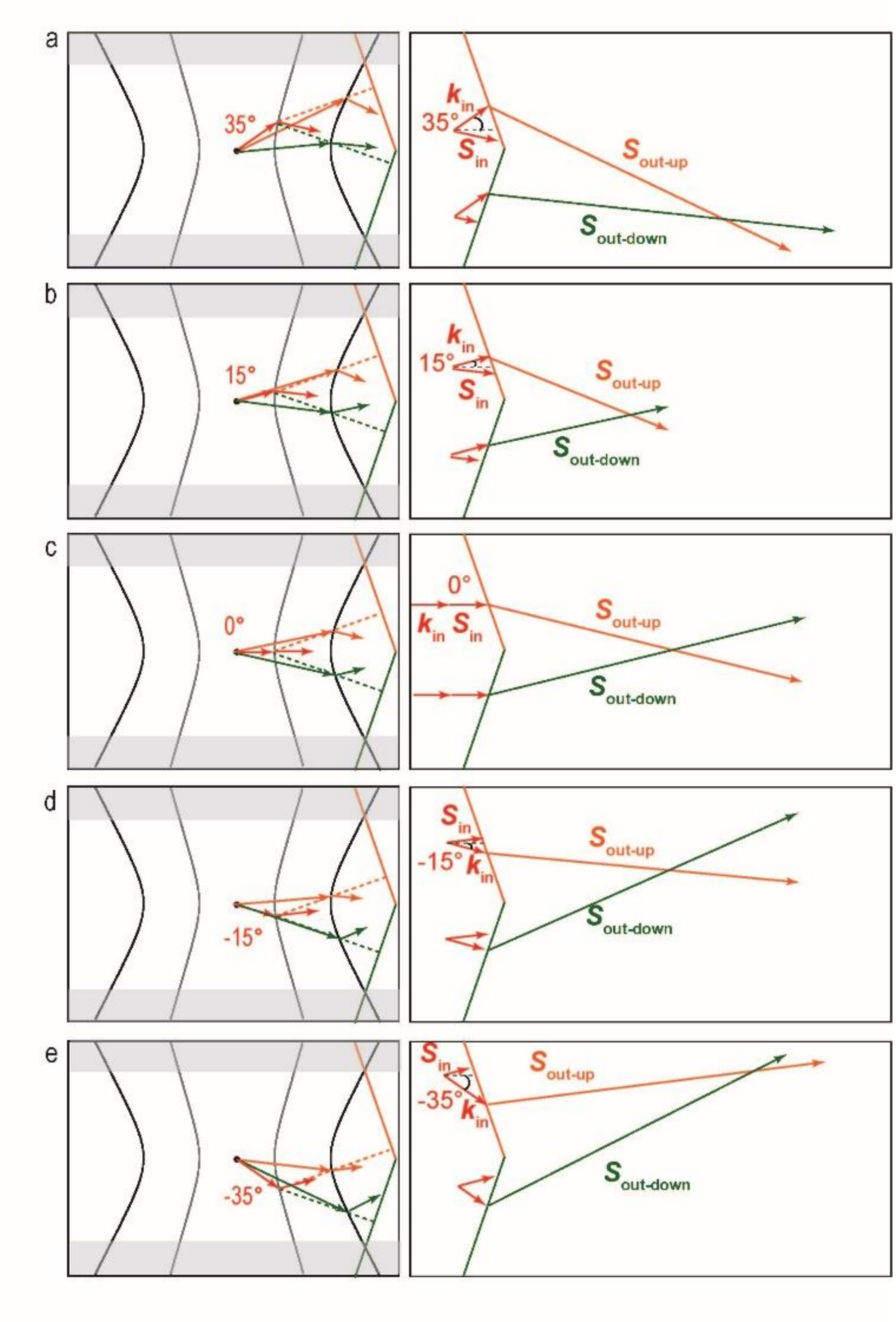

Supplementary Figure 11 Hyperlens with tilted incident polaritons with non-collinear *k* and *S*. a-e, Focusing of polaritons with non-collinear incident $\mathbf{k}_{\text{in}}$ and $\mathbf{S}_{\text{in}}$ at a representative tilting angle of 35º (a), 15º (b), 0º (c), -15º (d), and -35º (e) based on a semi-analytical approach. The boundary of the lens shown here is more open than the optimized lens in Fig. 4 of the main text. The refracted wavevectors $\mathbf{k}_{\text{out}}$ thus lay within the white areas, where the curvature of the IFCs shows a non-monotonical variation. This yields a complex trajectory (right column) of the focus spot with different tilt angle of $\mathbf{k}_{\text{in}}$. Grey and black curves in the left column represent IFCs of polaritons in the α-MoO_3/air and α-MoO_3/SiO_2 media, respectively

Supplementary Note 8: Reflection of polaritons in hyperbolic media.

Like refraction, reflection of polaritons in anisotropic media can be understood by considering the momentum conservation of the incident polaritons at a given boundary. In our work, there are two cases of reflection of polaritons considering the momenta of reflected polaritons are: very large (Supplementary Figure 12a) and relatively small (Supplementary Figure 12b). As shown in Supplementary Figure 12a (same image as Fig.2a), the wavevector of reflected polaritons (brown arrow) is much larger than that of incident (red arrow) and refracted (green arrow) polaritons, thus resulting in a pronounce damping, and therefore cannot be resolved in the experimental near-field image. We note that this effect is another peculiar aspect of reflection/refraction of polaritons in hyperbolic media, where they can possess much larger momenta than the incident waves.

On the other hand, the reflection plays an important role in the optical phenomena of polaritons in hyperbolic media when the reflected wavevector is relatively small (brown arrow in Supplementary Figure 12b) considering momentum conservation at the boundary (orange line in Supplementary Figure 12b). When the boundary is horizontal (boundary-2 in Fig. 4b), the modulus of wavevector of incident and reflected polaritons is identical due to the symmetric IFCs. Meanwhile, there is no refraction upon the horizontal boundary because of the absence of a $\boldsymbol{k_{out}}$ that crosses the second hyperbolic IFC in the α-MoO_3/SiO_2 medium.

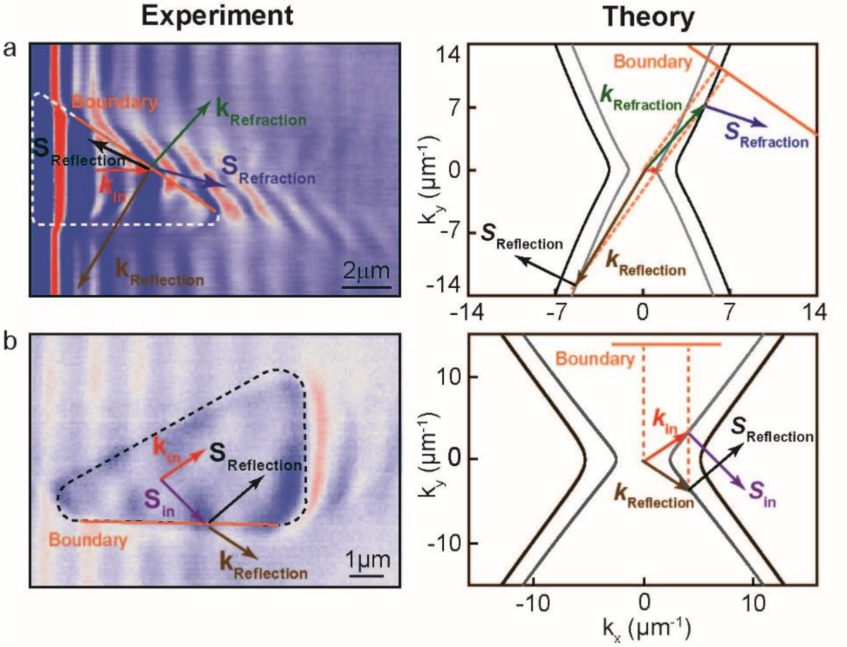

Supplementary Figure 12 Reflection of polaritons in hyperbolic media. a, Same experimental near-field image (left column) and analytic IFC (right column) as in Fig. 2a adding the reflection effect. As shown in the analytic IFCs, the momentum of reflected polaritons is much larger than those corresponding to the incident and refracted polaritons, making the fringes ascribed to reflected waves not visible in the experiments. **b**, the same experimental near-field image (left column) and analytic IFC (right column) as in Fig. 3c with a more detailed indication of reflection. As shown in the analytic IFCs, the momentum of reflected polaritons is identical to the one of

incident polaritons. The grey and black curves in the right column represent IFCs of polaritons in the α-MoO_3/air and α-MoO_3/SiO_2 media respectively

Supplementary Note 9: Analytical derivation of the shape of planar lenses in hyperbolic media.

In this section, organized in a tutorial style, we perform the analytical calculation of the optimal shape of a lens in hyperbolic media. In the first part, we consider a two-dimensional (2D) model, in which the lens is a curved boundary separating two different biaxial half-spaces. This case is introduced to illustrate our approach to engineering a hyperbolic lens. In the second part, we take a more realistic and rigorous approach, in which confined polaritons propagate in a thin film of a biaxial crystal, namely α-MoO_3.

1. Two dimensional case

In this part, we consider a system composed of two different biaxial half-spaces (without loss of generality, we will assume that they are infinite along the z direction) and separated by a surface whose shape is given by the equation f (x, y) = 0 (see Supplementary Figure 13a). We will assume that the dielectric permittivity tensors of both media have the following diagonal form:

$$\hat{\varepsilon}_i = \begin{pmatrix} \varepsilon_{ix} & 0 & 0 \\ 0 & \varepsilon_{iy} & 0 \\ 0 & 0 & \varepsilon_{iz} \end{pmatrix}, \qquad (1)$$

where $i = 1{,}2$ stands for the number of the medium. It means that we align the optics axes of the first crystal parallel to the corresponding optics axes of the second crystal and one of these axis parallel to the z direction. Also, x and y coordinate axes are chosen along the main crystal axes. A plane wave, polarized in the xy-plane and with wavevector **k** and Poynting vector **S**, is incident on the surface f (x, y) = 0. To mimic our experiment, we assume that this wave satisfies the conditions: $\mathbf{S} \parallel \mathbf{k} \parallel \mathrm{y}-$ axis. Therefore, the dispersion of an electromagnetic wave propagating in the first medium is given by Fresnel's equation[2]:

$$F\,(k_{1x}, k_{1y}) \equiv \frac{k_{1x}^2}{\varepsilon_{1y}} + \frac{k_{1y}^2}{\varepsilon_{1x}} - k_0^2 = 0, \qquad (2)$$

where $k_0 = \omega/c$, ω and c being the frequency of the wave and the speed of light, respectively. In lossless case, when dielectric permittivities are completely real, the Poynting vector is parallel to the group velocity. Therefore, **S** is given by $\mathbf{S}_1 \sim \nabla F\,(k_{1x}, k_{1y})$, or, more explicitly:

$$S_{1x} \sim \frac{k_{1x}}{\varepsilon_{1y}}; \quad S_{1y} \sim \frac{k_{1y}}{\varepsilon_{1x}}. \qquad (3)$$

When taking into account the optical losses of the medium, the direction of the Poynting vector will be slightly tilted with respect to the one predicted by Eq. (3), but we will neglect this effect here for the sake for simplicity, i.e. we will restrict ourselves to the lossless case. Let us consider now two different optical paths to the point (0,0): one along the y-axis and the other starting from an arbitrary point of the lens surface (labeled as "2" in Supplementary Figure 13a). The phase of the waves propagating along these paths are the following:

$$\Phi_{10} = -k_{2y}(0)(y-F) - k_{1y}(0)F = \sqrt{\varepsilon_{2x}}(y-F) + \sqrt{\varepsilon_{1x}}F; \qquad (4a)$$

$$\Phi_{20} = -k_{1y}(\varphi)y - k_{1x}(\varphi)x = -k_{1y}(\varphi)\left(y + \frac{x^2}{y}\frac{\varepsilon_{1y}}{\varepsilon_{1x}}\right), \qquad (4b)$$

where φ can be calculated geometrically as:

$$\tan\varphi = \frac{x}{y} = \frac{S_x}{S_y} = \frac{\varepsilon_{1x}k_{1x}}{\varepsilon_{1y}k_{1y}}. \qquad (5)$$

Therefore, we can immediately calculate $k_{1y}(\varphi)$ using Eq. (5) and Fresnel's equation, given by Eq. (2), according to:

$$k_{1y}(\varphi) = -\sqrt{\frac{\varepsilon_{1x}}{1+\frac{\varepsilon_{1y}x^2}{\varepsilon_{1x}y^2}}}. \qquad (6)$$

For these waves to focus at the point (0, 0), and, consequently, for the curve f (x, y) = 0 to be a hyperbolic refractive lens with focal distance F, both phases must be equal, i.e. $\Phi_{10} = \Phi_{20}$. Under this condition, the phases of all the waves coming through the lens to the point (0, 0) will be equal, as the point "2" is arbitrary. The shape of the lens is thus defined by:

$$\sqrt{\varepsilon_{1x}}F + \sqrt{\varepsilon_{2x}}(y-F) = \frac{\varepsilon_{1x}y}{\sqrt{\varepsilon_{1xy}^2+\varepsilon_{1y}x^2}}\left(y + \frac{\varepsilon_{1y}x^2}{\varepsilon_{1x}y}\right) = \sqrt{\varepsilon_{1x}y^2 + \varepsilon_{1y}x^2}. \qquad (7)$$

After some simplification, we obtain that the lens should be have a hyperbolic shape:

$$\left(y - \frac{\sqrt{\varepsilon_{2x}}}{\sqrt{\varepsilon_{1x}}+\sqrt{\varepsilon_{2x}}}F\right)^2 + \frac{\varepsilon_{1y}}{\varepsilon_{1x}-\varepsilon_{2x}}x^2 = \frac{\varepsilon_{1x}}{(\sqrt{\varepsilon_{1x}}+\sqrt{\varepsilon_{2x}})^2}F^2. \qquad (8)$$

To verify this result, we perform a full-wave numerical simulation of this system using the finite-element method COMSOL Multiphysics (see Methods). We chose $F = 3\mu m$, $\varepsilon_{1x} = 20 + 0.3i$, $\varepsilon_{1y} = -20 + 0.3i$, $\varepsilon_{2x} = 10 + 0.3i$, $\varepsilon_{2y} = -10 + 0.3i$. Supplementary Figure 13b-c show waves focusing at the (0, 0) point, in an excellent agreement with our theoretical prediction.

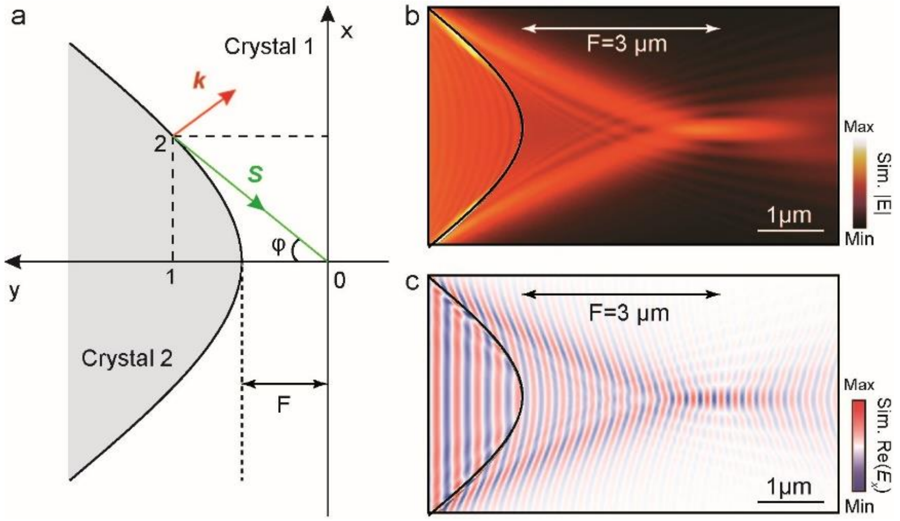

Supplementary Figure 13 Analytic derivation of lens shape between two different biaxial half-spaces. a, Schematics of the lens between two different biaxial half-spaces: crystal 1 (white

region) and crystal 2 (grey region). **b-c,** Distribution of the absolute value and the real part of the x-component of the electric field respectively, based on full-wave numerical simulations.

2. Thin film case

However, the goal of this work is not the lensing of free-space light, but the lensing of highly confined polaritons propagating in finite-thickness-slab of biaxial crystal. Therefore, let us consider now a homogeneous biaxial slab with thickness *d* embedded between two different semi-infinite isotropic media. In other words, we consider a system which is similar to the one shown in Supplementary Figure 13a, but with the same biaxial film and superstrate in both regions, and with different substrates (Supplementary Figure 14a). In this setting, we approximate the slab by a thin-film with vanishing thickness $d \rightarrow 0$. The limit of vanishing slab thickness is of a great practical interest and has been already used for the analysis of hyperbolic polaritons in thin slabs of α-MoO_3[3,4]. Under this limit, we assume that all the components of the dielectric tensor $\hat{\varepsilon}$ are large, i.e.$|\varepsilon_i| \gg 1$ $(i = x, y, z)$ and approximate the slab of finite thickness by a 2D conductive sheet, with effective conductivity $\hat{\sigma}$ given by $\hat{\sigma} = \omega d\hat{\varepsilon}/4\pi i$. Moreover, we consider waves with high momentum, $k \gg k_0$, which is justified as we are studying highly confined polaritons. Under such approximations, the dispersion of polaritons propagating in the film is given by[4]:

$$F\left(q_x, q_y\right) = iq\left(\alpha_x \cos^2\varphi + \alpha_y \sin^2\varphi\right) + \varepsilon = 0, \qquad (9)$$

where $\mathbf{q} = \mathbf{k}/k_0$ is the normalized wavevector, and $q = \sqrt{q_x^2 + q_y^2}$. Furthermore, $\alpha = \frac{2\pi}{c}\sigma = \frac{k_0 d\varepsilon}{2i}$ is the normalized effective 2D conductivity of the film, and $\varepsilon_{1,2}$ is an arithmetical mean of the substrate and superstrate dielectric permittivities. Eq. (9) can also be written as:

$$k_0 d\left(\varepsilon_x q_x^2 + \varepsilon_y q_y^2\right) + 2\varepsilon q = 0. \qquad (10)$$

In analogy to the previous section, we derive the direction of the Poynting vector as $\boldsymbol{S}_1 \sim \nabla F\left(k_{1x}, k_{1y}\right)$:

$$S_{1x} \sim q_{1x}\left(\varepsilon_x + \frac{\varepsilon_1}{qk_0 d}\right); \quad S_{1y} \sim q_{1y}\left(\varepsilon_y + \frac{\varepsilon_1}{qk_0 d}\right). \qquad (11)$$

Finally, we impose that the polaritonic phase along two different optical paths must be equal, i.e. $\Phi_{10} = \Phi_{20}$, yielding:

$$\Phi_{10} = -k_{2y}(0)(y - F) - k_{1y}(0)F = -\frac{2\varepsilon_2}{k_0 d\varepsilon_y}(y - F) - \frac{2\varepsilon_1}{k_0 d\varepsilon_y}F; \qquad (12a)$$

$$\Phi_{20} = -k_{1y}(\varphi)y - k_{1x}(\varphi)x. \qquad (12b)$$

Combining Eqs. (10), (11) and (12) with the relation $\frac{x}{y} = \frac{S_x}{S_y}$, we obtain a system of equations which defines the shape of the lens:

$$-\frac{2\varepsilon_1}{\varepsilon_y}F - \frac{2\varepsilon_2}{\varepsilon_y}\,(\mathrm{y} - F) = \frac{\kappa\left(\varepsilon(x^2+y^2)+\kappa\left(\varepsilon_y x^2+\varepsilon_x y^2\right)\right)}{\sqrt{\left(\varepsilon_1+\varepsilon_y\kappa\right)^2 x^2+(\varepsilon+\varepsilon_x\kappa)^2 y^2}}; \qquad (13)$$

$$\kappa = 2\varepsilon_1 \frac{(\varepsilon_x\kappa+\varepsilon)^2 y^2+(\varepsilon_y\kappa+\varepsilon)^2 x^2}{\varepsilon_y(\varepsilon_x\kappa+\varepsilon)^2 y^2+\varepsilon_x(\varepsilon_y\kappa+\varepsilon)^2 x^2}, \qquad (14)$$

with $\kappa = qk_0 d$. To plot the shape of the lens, one should solve the system (13-14) numerically.

To verify our analytical definition of the shape of the lens, we perform full-wave numerical simulations. As a representative example, we consider a 100-nm-thick α-MoO_3 film[5] on top of SiO_2 (white region in Supplementary Figure 14) and air (grey region in Supplementary Figure 14), both covered by air. The simulation was performed at $\lambda_0 = 11.11\,\mu m$ and $F = 3\,\mu m$. Supplementary Figure 14 b-c show a good agreement with the theoretically predicted focal point.

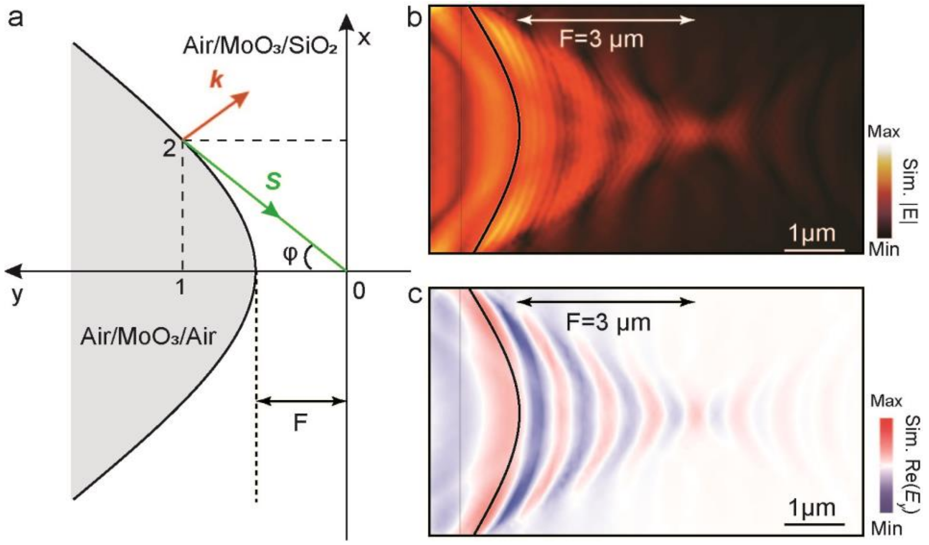

Supplementary Figure 14 Analytic derivation of lens shape between two hyperbolic media. a, Schematics of the lens between two hyperbolic media: Air/MoO_3/SiO_2 (white region) and Air/MoO_3/Air (grey region). **b-c,** Distribution of the absolute value and the real part of the y-component of the electric field respectively, based on full-wave numerical simulations.

Supplementary References

1. Castellanos-Gomez, A. *et al.* Deterministic transfer of two-dimensional materials by all-dry viscoelastic stamping. *2D Mater.* **1**, 011002 (2014).

2. Landau, L.D. *et al.* Electrodynamics of continuous media. Elsevier, **8** (2013).

3. Ma, W. *et al.* In-plane anisotropic and ultra-low-loss polaritons in a natural van der Waals crystal. *Nature* **562**, 557-562 (2018).

4. Álvarez-Pérez, G. *et al.* Analytical approximations for the dispersion of electromagnetic modes in slabs of biaxial crystals. *Phys. Rev. B* **100**, 235408 (2019).

5. Álvarez-Pérez, G. *et al.* Infrared Permittivity of the Biaxial van der Waals Semiconductor α-MoO_3 from Near- and Far-Field Correlative Studies. *Adv. Mater.* **32**, 1908176 (2020).
